# Neuronal Transmission of Subthreshold Periodic Stimuli Via Symbolic Spike Patterns

**DOI:** 10.3390/e22050524

**Published:** 2020-05-05

**Authors:** Maria Masoliver, Cristina Masoller

**Affiliations:** Departament de Física, Universitat Politècnica de Catalunya, St. Nebridi 22, 08222 Terrassa, Barcelona, Spain; maria.masoliver@gmail.com

**Keywords:** neural coding, neural noise, inter-spike intervals, spike trains, FitzHugh–Nagumo model, symbolic analysis, ordinal analysis, permutation entropy, mutual information, time series analysis

## Abstract

We study how sensory neurons detect and transmit a weak external stimulus. We use the FitzHugh–Nagumo model to simulate the neuronal activity. We consider a sub-threshold stimulus, i.e., the stimulus is below the threshold needed for triggering action potentials (spikes). However, in the presence of noise the neuron that perceives the stimulus fires a sequence of action potentials (a spike train) that carries the stimulus’ information. To yield light on how the stimulus’ information can be encoded and transmitted, we consider the simplest case of two coupled neurons, such that one neuron (referred to as neuron 1) perceives a subthreshold periodic signal but the second neuron (neuron 2) does not perceive the signal. We show that, for appropriate coupling and noise strengths, both neurons fire spike trains that have symbolic patterns (defined by the temporal structure of the inter-spike intervals), whose frequencies of occurrence depend on the signal’s amplitude and period, and are similar for both neurons. In this way, the signal information encoded in the spike train of neuron 1 propagates to the spike train of neuron 2. Our results suggest that sensory neurons can exploit the presence of neural noise to fire spike trains where the information of a subthreshold stimulus is encoded in over expressed and/or in less expressed symbolic patterns.

## 1. Introduction

Sensory neurons use sequences of electrical pulses (known as action potentials or spikes) to encode and transmit information of external temporal stimuli [1]. Any kind of perception results in sequences of spikes which the central nervous system processes. For example, when we touch, the central nervous system encodes the change of pressure in our skin as a sequence of spikes (or spike trains) fired by sensory neurons; when we hear, the central nervous system encodes the incoming pressure waves as spike trains fired by auditory neurons.

Neural coding is the research field that studies the relationship between external stimuli and neuronal responses. While the mechanisms underlying neural coding are not yet fully understood, it is known that neurons use different mechanisms to encode external stimuli, which are complementary (e.g., used simultaneously to represent different variables) or functional under different situations (e.g., depending on the frequency content of the signal, or on the signal-to-noise ratio, a rate code (based on the rate of the spikes) or a temporal code (based on the timing of the spikes) might be used) [2,3]. An important issue is how neural noise (stochastic electrical fluctuations which do not carry any information) affects the neural code [4,5].

Here we focus on the neuronal response to a weak periodic external input in the presence of neural noise. The signal is weak enough to be sub-threshold, i.e., by itself it does not induce spikes. However, neural noise triggers spikes, and in spite of the fact that the spikes are triggered by noise, the spike train contains information of the underlying weak signal. In this situation stochastic resonance has been observed, by which an appropriated level of noise enhances the regularity of the spike train [6,7].

Here we consider an alternative encoding mechanism, which is based on the frequency of occurrence of symbolic spike patterns that are defined by the temporal structure of the sequence of inter-spike intervals (ISIs) in a spike train. This approach to define symbolic patterns is known as ordinal analysis [8] and has been extensively used in various fields of research (see, e.g., [9,10] and references therein). The main advantages of this methodology are that it can be applied to raw, unprocessed data and is rather unaffected by the presence of noise, outliers or missing data [11]. However, only few studies have applied ordinal analysis to neuronal ISI sequences [12,13,14,15,16]. From the analysis of ISI sequences simulated with neuronal models, these studies have shown that either single neurons or coupled neurons, when they perceive a weak periodic stimulus, they respond by firing spike trains that have symbolic patterns whose frequencies of occurrence depend on the stimulus’s characteristics (amplitude and period).

Our aim is to determine whether the stimulus’s information, encoded in the spike train as over expressed and/or less expressed symbolic patterns, can propagate to another neuron that does not perceive the stimulus. We perform simulations of the well-known FitzHugh–Nagumo model [17,18]. We consider the simplest situation: two coupled neurons, such that one neuron (referred to as neuron 1) perceives a subthreshold periodic signal but the second neuron (neuron 2) does not perceive the signal. We show that, for appropriated parameters, both neurons fire spike trains that have symbolic patterns, whose frequencies of occurrence are similar for both neurons (i.e., both spike trains contain the same over expressed and/or less expressed patterns). Since the over expressed and less expressed patterns (patterns whose probability of occurrence is higher or lower than the probability expected in a uniform distribution) depend on the signal’s amplitude and period, the information that is encoded in the spike train of neuron 1 propagates to the spike train of neuron 2.

This paper is organized as follows. Section 2 presents the FitzHugh–Nagumo model and the methods used to analyze the spike trains, Section 3 presents the results and Section 4 presents the discussion.

## 2. Model and Methods

### 2.1. Model

We consider two coupled FitzHugh–Nagumo neurons, with a periodic signal applied to neuron 1:

(1) $$\begin{array}{ccc} \epsilon_{1}\dot{u_{1}} & = & u_{1}-\frac{u_{1}^{3}}{3}-v_{1}+a_{0}\cos\left(2\pi t/T\right)+\sigma \left(u_{2}-u_{1}\right)+\sqrt{2D_{1}}\xi_{1}\left(t\right),\\ \dot{v_{1}} & = & u_{1}+a_{1},\\ \epsilon_{2}\dot{u_{2}} & = & u_{2}-\frac{u_{2}^{3}}{3}-v_{2}+\sigma \left(u_{1}-u_{2}\right)+\sqrt{2D_{2}}\xi_{2}\left(t\right)\\ \dot{v_{2}} & = & u_{2}+a_{2} \end{array}$$

The dimensionless variables $u_{i}$ and $v_{i}$ are a fast variable that represents the voltage of the membrane, and a slow recovery-like variable that represents the refractory properties of the membrane; $a_{1}$, $a_{2}$, $\epsilon_{1}$ and $\epsilon_{2}$ are parameters that control the spiking activity of the uncoupled neurons. The terms $\sigma (u_{2}-u_{1})$ and $\sigma (u_{1}-u_{2})$ mimic direct gap junction coupling that is linear, diffusive and mutual. $a_{0}$ and *T* are the signal’s amplitude and period respectively. Neural noise is modeled with independent Gaussian white noise terms, $\langle \xi_{i}\left(t\right)\xi_{j}\left(t^{\prime }\right)\rangle =\delta \left(i-j\right)\delta \left(t-t^{\prime }\right)$, and $D_{1}$ and $D_{2}$ are the noise levels.

### 2.2. Methods

#### 2.2.1. Simulations

Unless specifically stated, the neurons have identical parameters: $a_{1}=a_{2}=a=1.05$, $\epsilon_{1}\;=\;\epsilon_{2}=\epsilon =0.01$ and $D_{1}=D_{2}=D=5\times 10^{-6}$; for these parameters in the absence of noise, signal or coupling (D=0, $a_{0}=0$, σ=0), there is no activity and the neurons stay in the rest state. The coupling strength, σ, and the signal parameters, $a_{0}$ and *T*, are varied. $a_{0}$ and *T* are chosen such that the signal is subthreshold, i.e., without noise or coupling the signal does not induce spikes but small sinusoidal oscillations.

The model equations are integrated, starting from random initial conditions, using the Euler–Maruyama method with an integration step of $dt=10^{-3}$. For each set of parameters, the voltage-like variables, $u_{1}$ and $u_{2}$, are analyzed. A spike is detected at time $t_{i}^{1,2}$ whenever $u_{1,2}\left(t_{i}^{1,2}\right)=0$ considering only the ascensions. The simulation finishes when $10^{5}$ spikes are detected in both neurons. Then, for each neuron, the ISI sequence is computed, $\left\{I_{i}^{1,2};I_{i}^{1,2}=t_{i+1}^{1,2}-t_{i}^{1,2}\right\}$ with $t_{i}^{1}$ ($t_{i}^{2}$) being the time of the *i*th spike of neuron 1 (of neuron 2).

#### 2.2.2. Ordinal Patterns

The symbolic method of time series analysis known as *ordinal analysis* [8] is used to analyze the ISI sequences extracted from the two neurons’ spike trains. In this approach the actual ISI values $\left\{I_{1},\ldots ,I_{i},\ldots ,I_{N}\right\}$ are not taken into account, instead, their relative temporal ordering is considered: each ISI sequence is transformed into a sequence of ordinal patterns that are defined by the relative order of *L* consecutive ISI values. For each interval $I_{i}$ the subsequent L−1 intervals are considered and compared. The total number of possible order relations (i.e., ordinal patterns of length *L*) is equal to the number of permutations L!. If we set L=2 we have only two patterns: 01 and 10 for $I_{1}<I_{2}$ and $I_{1}>I_{2}$, respectively; for L=3, we have 3! = 6 possible patterns, which are listed in Table 1.

The symbolic sequence of ordinal patterns is computed using the function perm_indices defined in [19]. It has been recently shown that when a time series contains a large number of equal values (in our case, equal ISIs), they can give rise to false conclusions regarding the presence of temporal structures [20]. To avoid this problem, when two ISIs are equal, a very small random term is added to one of the ISIs before the function perm_indices is used to determine the label of the pattern.

Then, the frequency of occurrence of the different patterns (the ordinal probabilities) are estimated as $p_{i}=N_{i}/M$ where $N_{i}$ is the number of times the i-th pattern occurs in the sequence, and *M* is the total number of patterns. If all the patterns are equiprobable there is no temporal structure in the spike sequence. On the other hand, more frequent (over expressed) or infrequent (under expressed) patterns uncover temporal structures in the spike sequence. A binomial test is used to address the statistical significance of over or less expressed patterns: if $p_{i}\in \left[p-3\sigma_{p},p+3\sigma_{p}\right]\forall i$ (with p=1/L! and $\sigma_{p}=\sqrt{p(1-p)/M}$), the probabilities are consistent with the uniform distribution, else, if there is at least one $p_{i}\notin \left[p-3\sigma_{p},p+3\sigma_{p}\right]$, then there are statistically significant deviations from the uniform distribution that uncover the presence of over expressed and/or less expressed patterns.

Here we use L=3 and L=4 which allow to detect order relations among three or four consecutive ISI (i.e., four or five consecutive spikes). This choice is motivated by the fact that using a larger *L* value means that there is a large number of probabilities to calculate, which is computationally expensive as extremely long simulations (containing a very large number of spikes) will be required in order to have a reliable estimation of the ordinal probabilities (see [12] for a discussion of the data requirements).

#### 2.2.3. Time Series of Ordinal Patterns

**Figure 1:**
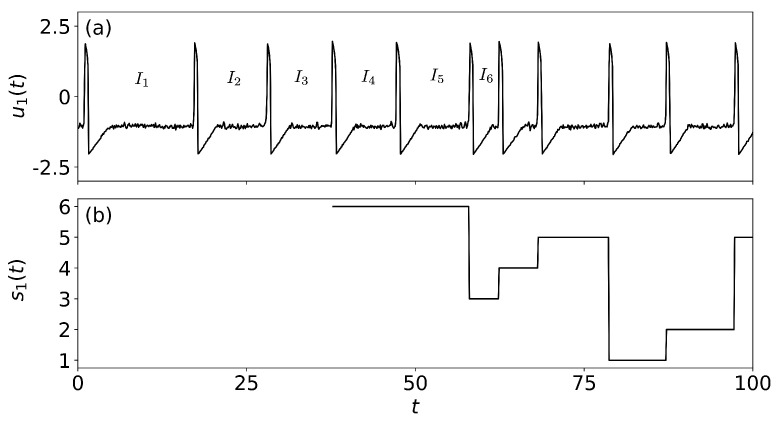
(**a**) Time series of the voltage-like variable $u_{1}\left(t\right)$ of neuron 1 and (**b**) the corresponding ordinal time series $s_{1}\left(t\right)$. The first three ISIs define, at the time of the 4th spike, the pattern 210 (label 6 according to Table 1) because $I_{1}>I_{2}>I_{3}$. When the 5th spike occurs, the pattern is defined in terms of $\left\{I_{2},I_{3},I_{4}\right\}$, and is still pattern label 6 because $I_{2}>I_{3}>I_{4}$. After the 6th spike, the pattern changes to label 3 (102) because the order of $\left\{I_{3},I_{4},I_{5}\right\}$ is $I_{5}>I_{3}>I_{4}$. The next pattern is label 4 (120) because $I_{5}>I_{4}>I_{6}$. The parameters are $a_{0}=0.05$, T=10, and σ=0.05.

While the analysis of the ordinal probabilities reveals the presence of over expressed and/or under expressed patterns in the ISI sequences, it does not provide information about the temporal order in which the different patterns appear in the ISI sequence. To overcome this limitation we define, for each neuron, a time series of the ordinal patterns, $s_{1,2}\left(t\right)$, which keeps track of the pattern at each time step. An example of $u_{1}\left(t\right)$ and, for L=3, the corresponding $s_{1}\left(t\right)$ is presented in Figure 1. The temporal order among the first three ISIs, $\left\{I_{1},I_{2},I_{3}\right\}$, defines the first ordinal pattern at the time of occurrence of the 4th spike. This first ordinal pattern is kept along time until a new spike occurs, then the second ordinal pattern will be defined with the sequence $\left\{I_{2},I_{3},I_{4}\right\}$ and will be kept along time until a new spike occurs. This procedure is repeated until the end of the simulation, thus, for each u(t) we obtain an associated sequence of integer numbers, s(t)∈[1,L!], that has almost the same length as u(t) (the difference being the initial time during which there are not enough spikes to define a pattern).

#### 2.2.4. Information Measures

To quantify the amount of information contained in the ISI sequence extracted from the spike train of a neuron, we transform the ISI sequence in a sequence of ordinal patterns, and use the normalized permutation entropy [8]:

(2) $$H=-\frac{\sum_{i}p_{i}\log p_{i}}{\log L!}.$$

*H* ranges between 0 ($p_{i}=1,p_{j}=0$ ∀j≠i, i.e., only one pattern is expressed in the ISI sequence) and 1 ($p_{i}=1/L!$ ∀i, i.e., all patterns are equally expressed in the ISI sequence). It is worth noticing that if the spike train is perfectly regular (i.e., all ISIs are equal) because, as explained before, as very small random term is added to one of the ISIs before determining the label of the pattern, in this situation all the patterns will be equally expressed and therefore H=1. Thus, H=1 corresponds to either a ISI sequence with no temporal structure, or to a perfectly regular ISI sequence. Nevertheless, small deviations from H=1 can be used to identify the presence of temporal structures in the ISI sequence.

To quantify the amount of information shared by the spike trains of the two neurons we use the mutual information [21]:

(3) $$MI=H_{1}+H_{2}-H_{12},$$

computed for the time series of ordinal patterns, $s_{1}\left(t\right)$ and $s_{2}\left(t\right)$, extracted from the spike trains of neurons 1 and 2. $H_{1}$ and $H_{2}$ are the entropies of $s_{1}\left(t\right)$ and $s_{2}\left(t\right)$, and $H_{12}$ is the joint entropy

(4) $$H_{12}=-\frac{\sum_{i}\sum_{j}p_{ij}^{12}\log p_{ij}^{12}}{\log L!},$$

where $p_{ij}^{12}$ is the probability that the i-th pattern occurs in the spike train of neuron 1 at the same time as the j-th pattern occurs in the spike train of neuron 2, i.e., the probability that $s_{1}\left(t\right)=i$ and $s_{2}\left(t\right)=j$. If $s_{1}\left(t\right)$ and $s_{2}\left(t\right)$ are independent, $p_{ij}^{12}=p_{i}p_{j}$, $H_{12}=H_{1}+H_{2}$, and thus MI=0 (in the limit of a very long time series). On the contrary, if both time series are identical, $p_{ii}^{12}=p_{i}$ and $p_{ij}^{12}=0$ ∀j≠i. In this case $H_{12}=H_{1}=H_{2}$ and thus, $MI=H_{1}=H_{2}$.

To complement the symbolic analysis, we also calculate the cross-correlation coefficient between the time series of the voltage-like variable of both neurons, $u_{1}\left(t\right)$ and $u_{2}\left(t\right)$:

(5) $$CC=\frac{\left(u_{1}-\langle u_{1}\rangle \right)\left(u_{2}-\langle u_{2}\rangle \right)}{\sqrt{\langle u_{1}^{2}\rangle -{\langle u_{1}\rangle }^{2}}\sqrt{\langle u_{2}^{2}\rangle -{\langle u_{2}\rangle }^{2}}}.$$

CC quantifies the degree of linear synchronization of the two spike trains: |CC|=0 if the spike trains are uncorrelated, while |CC|=1 if they are identical.

## 3. Results

**Figure 2:**
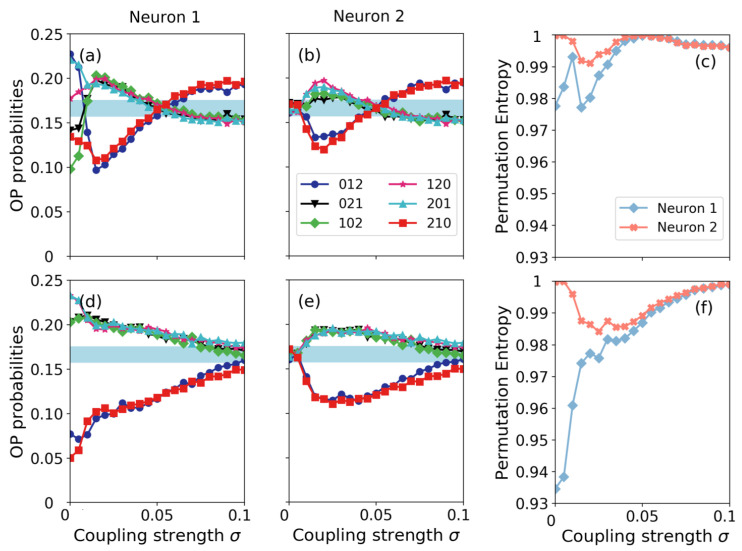
Ordinal probabilities extracted from the spike train of neuron 1 [(**a**) and (**d**)] and of neuron 2 [(**b**) and (**e**)] and their permutation entropy [(**c**) and (**f**)] as a function of the coupling strength, σ. The amplitude of the signal is $a_{0}=0.07$ and the period is T=8 [(**a**)–(**c**)] and T=10 [(**d**)–(**f**)].

Figure 2 displays the ordinal pattern (OP) probabilities and the permutation entropies of the two neurons as a function of the coupling strength for two periods of the external signal: in panels (a), (b) and (c) T=8; in panels (d), (e) and (f) T=10. In panels (a) and (d) we see that the ordinal probabilities computed for neuron 1 are different for T=8 or T=10. As explained in the Introduction, for the spike train of the neuron that perceives the signal (neuron 1), the ordinal probabilities depend on the period and amplitude of the signal [12,13]. Regarding the probabilities computed from the spike train of neuron 2, we see in panels (b) and (e) that, if the neurons are only weakly coupled, all the probabilities are within the gray region that represents values consistent with the uniform distribution. This is expected because, if the coupling strength is too weak, neural noise dominates the spiking activity of neuron 2. However, above a certain value of the coupling strength, the ordinal pattern probabilities of neuron 2 separate from the equi-probable region, and we see that the two neurons have the same over-expressed and under-expressed patterns, which indicates that the signal information propagates from neuron 1 to neuron 2. For both periods, above σ=0.05 the ordinal pattern probabilities and the permutation entropy are almost equal for both neurons, which suggests that the activity of the neurons is synchronized.

**Figure 3:**
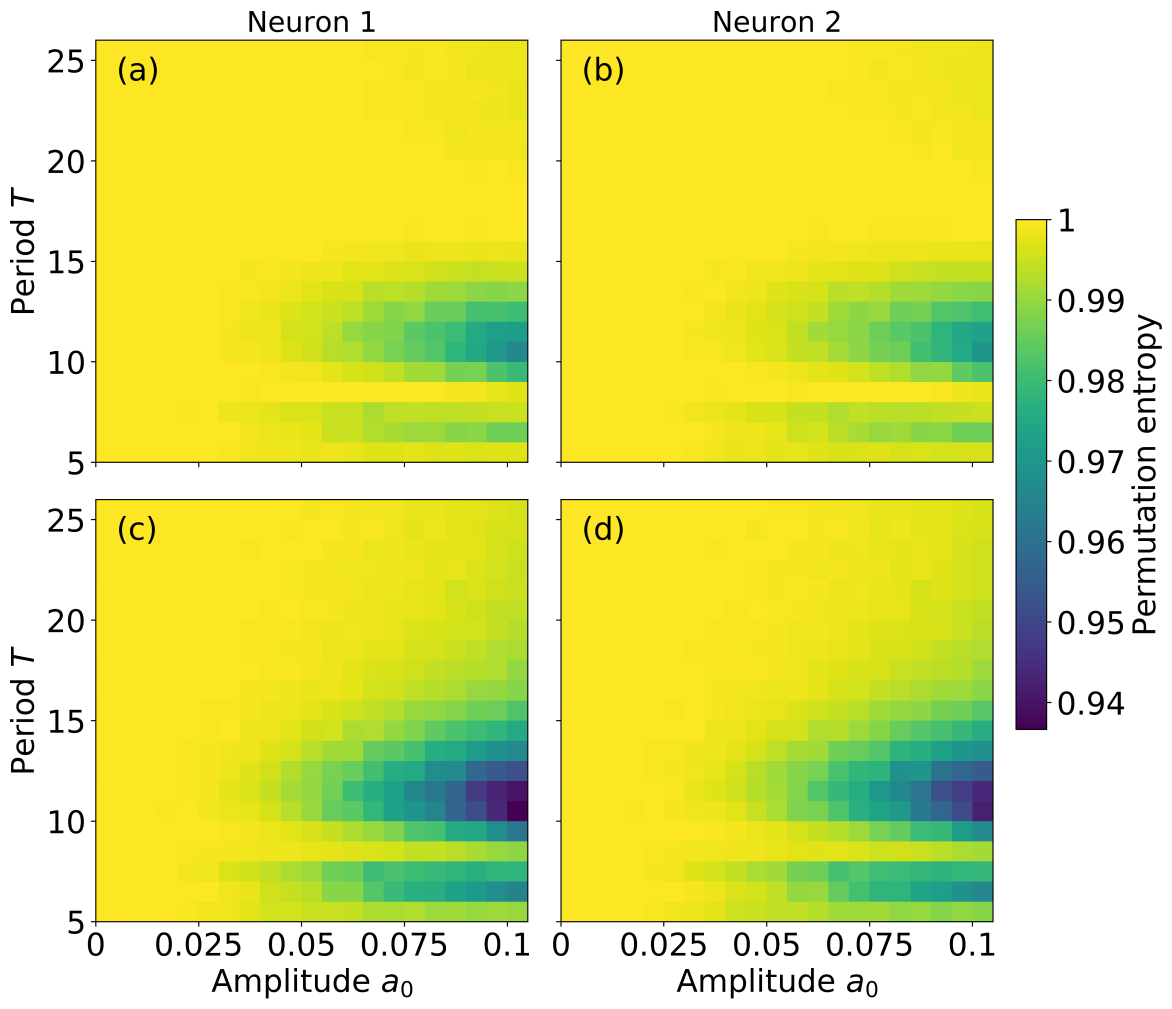
Permutation entropy of neuron 1 [(**a**) and (**c**)] and of neuron 2 [(**b**) and (**d**)] as a function of the amplitude and period of the signal. The coupling strength is σ=0.05. In (**a**), (**b**) L=3; in (**c**), (**d**) L=4.

To investigate the role of the signal’s parameters, $a_{0}$ and *T*, we calculate the permutation entropy for neuron 1 and neuron 2, varying $a_{0}$ and *T* while keeping constant the coupling strength (σ=0.05). $a_{0}$ and *T* are limited to the range in which the signal is subthreshold (for $a_{0}>0.1$ or T<5 the signal is suprathreshold). Figure 3 displays the results: $H_{1}$ and $H_{2}$ are shown in color code, in the left and right columns respectively. To check the robustness of the results, we use ordinal patterns of length L=3 (top row) and L=4 (bottom row). We observe that $H_{1}$ and $H_{2}$ have similar values for all $a_{0}$ and *T*, which suggests that the neurons are synchronized. We also note that, if the signal amplitude, $a_{0}$, is large enough, and its period is not too fast nor too slow: $H_{1}$ and $H_{2}$ are <1. This occurs when $a_{0}>0.05$ and T∈[5−15], which corresponds to about one to three times the mean ISI (for the parameters in Figure 3 and $a_{0}=0$, $\langle I\rangle =5.53$ for both neurons). When $H_{1}$ and $H_{2}$ are <1 there are over and/or under expressed patterns in the ISI sequences, which means that the signal is encoded and transmitted. However, we note that there is an almost horizontal yellow line for T∼8 that indicates $H_{1},H_{2}∼1$. This means that, if the period of the weak signal is T∼8, the signal is not codified. This can be understood by inspecting the probabilities shown in Figure 2a,b: we see that for σ<0.05 patterns 201, 021, 120 and 102 are over-expressed, while for σ>0.05 patterns 012 and 210 are over-expressed; for σ≈0.05 there is a cross over and the probabilities of all the patterns are similar.

**Figure 4:**
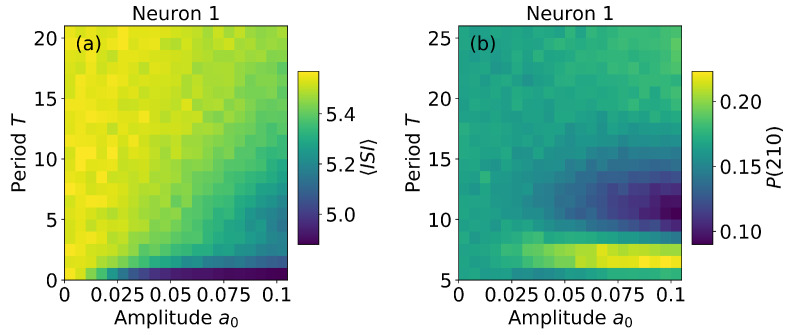
Mean inter-spike-interval, $\langle ISI\rangle$ (**a**), and the probability of pattern 210, P(210) (**b**), computed from the spike train of neuron 1, as a function of the amplitude and the period of the signal. We see that $\langle ISI\rangle$ is nearly constant, but pattern 210 is over expressed or under expressed depending on the signal’s period. The coupling strength is σ=0.05.

To complement the analysis we compare two mechanisms for encoding the signal information: the average firing rate and the frequency of occurrence of an ordinal pattern. As the trend pattern 210 has often asymmetric abundance (is either over or under expressed), we analyze the frequency of occurrence of this pattern and compare with the average inter-spike-interval, $\langle ISI\rangle$. Figure 4 displays $\langle ISI\rangle$ (panel a) and P(210) (panel b) computed from the spike train of neuron 1 (very similar plots are obtained for neuron 2, not shown). We see that $\langle ISI\rangle$ has only a small variation (of about 10%) because it mainly depends on the level of noise; in contrast, in the region $a_{0}>0.05$ and T<15, pattern 210 is over expressed or under expressed depending on the value of *T*. This suggests that, for subthreshold periodic signals in the presence of noise, the frequency of particular spike patterns can carry the signal’s information, which can not be encoded in the firing rate that is determined by the level of noise.

**Figure 5:**
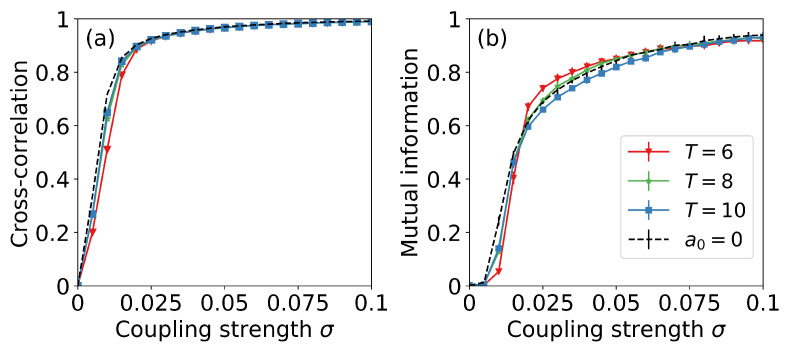
Cross-correlation (**a**) and mutual information (**b**) as a function of coupling strength without external signal and with a signal of amplitude $a_{0}=0.07$ and different periods indicated in the inset.

In order to confirm the synchronized activity of the two neurons, we calculated the cross-correlation of the fast variables of neurons 1 and 2, $u_{1}\left(t\right)$ and $u_{2}\left(t\right)$, and also, the mutual information of the ordinal sequences derived from them, $s_{1}\left(t\right)$ and $s_{2}\left(t\right)$. Figure 5 displays the CC value [panel (a)] and the MI value [panel (b)], as a function of the coupling strength, without signal and with a signal of amplitude $a_{0}=0.07$ and different periods. To investigate the variability of these values, we divided the time series in 30 segments and computed CC and MI in each segment. The error bars obtained were, in most cases, smaller than the plot markers (not shown).

The cross-correlation grows almost linearly with the coupling strength, and indicates a strong synchronization already for σ=0.025 (CC∼0.98). The mutual information shows an abrupt increase at a coupling value that depends on the period of the signal. The cross-correlation is rather independent of the period of the signal for all the range of coupling strengths studied; in contrast, the mutual information, for an intermediate range of coupling (0.02<σ<0.075), captures small differences that depend on the period. It would be interesting to compare the MI results with those obtained with other synchronization quantifiers, such as the phase locking value [22] or the ordinal synchronization measure that was recently introduced in Ref. [23]; however, this analysis is left for future work.

**Figure 6:**
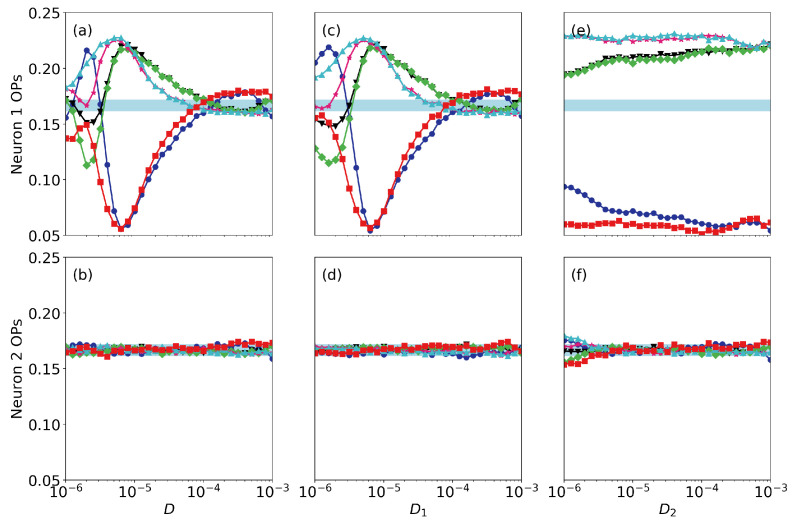
Ordinal probabilities (OPs) as a function of the noise level when the coupling strength is σ=0.005. Panels (**a**), (**c**), (**e**) [(**b**), (**d**), (**f**)] display the probabilities computed from the spike train of neuron 1 [neuron 2]. In (**a**), (**b**) the noise level is the same for both neurons; in (**c**), (**d**) we vary the noise level of neuron 1, keeping fixed $D_{2}=5\times 10^{-6}$; in (**e**), (**d**) we vary the noise level of neuron 2, keeping fixed $D_{1}=5\times 10^{-6}$. The signal’s parameters are $a_{0}=0.07$ and T=10.

**Figure 7:**
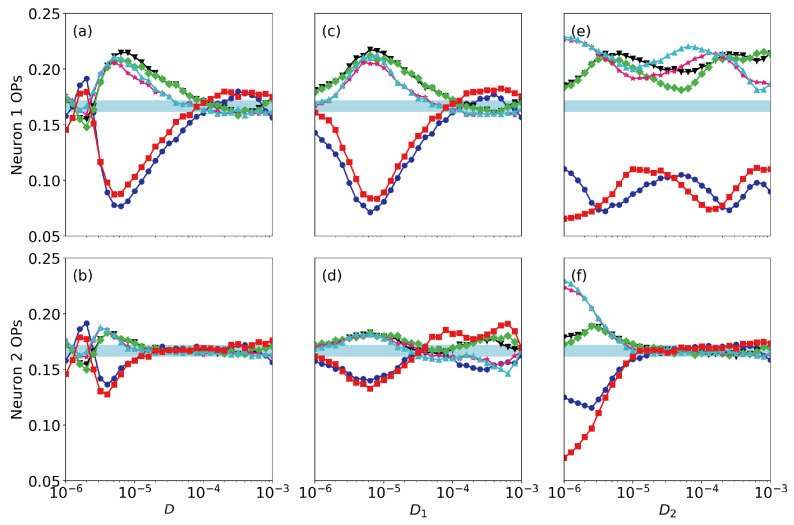
As Figure 6 but when the coupling strength is σ=0.01.

**Figure 8:**
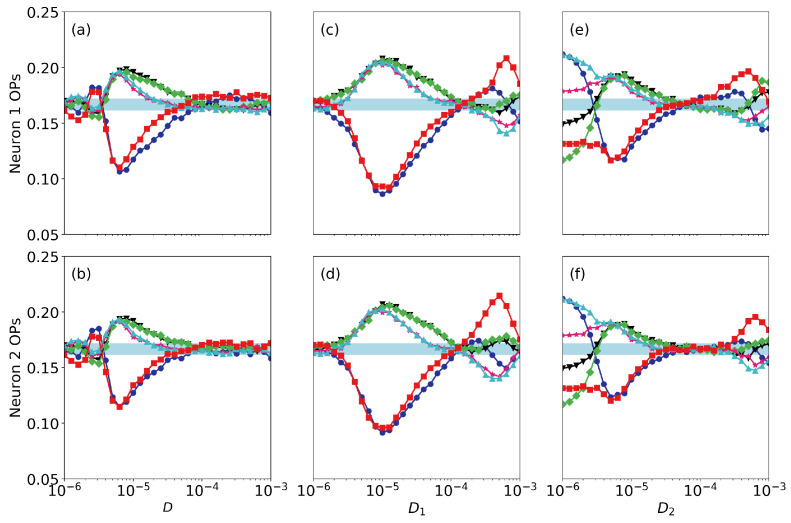
As Figure 6 but when the coupling strength is σ=0.05.

**Figure 9:**
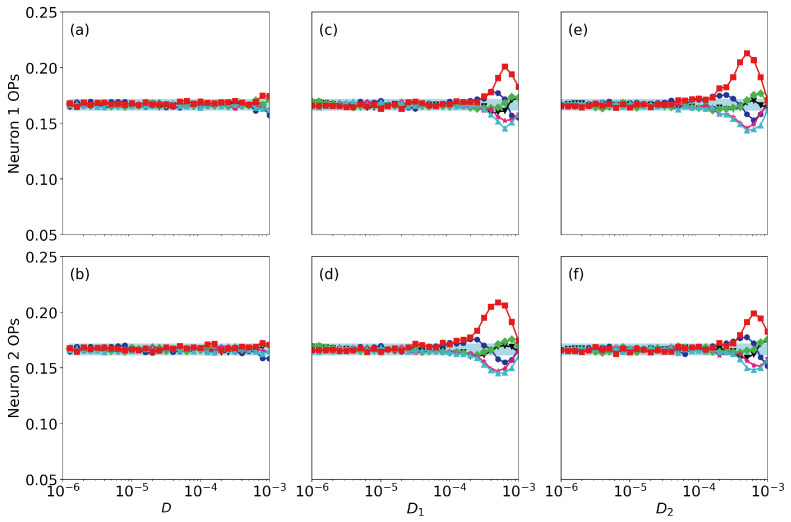
As Figure 8 but when there is no external signal, $a_{0}=0$.

Finally, we address the roles of noise and of small differences in the neurons’ parameters. Figure 6 displays the ordinal probabilities vs. the level of noise, when it is the same in both neurons [panels (a), (b)], when we vary the noise level of neuron 1 (that perceives the signal), $D_{1}$, keeping fixed the noise level of neuron 2, $D_{2}$, [panels (c), (d)] and when we vary $D_{2}$, keeping fixed $D_{1}$ [panels (e), (f)]. Here the coupling strength is very small (σ=0.005) and we can see that the spike train of the second neuron does not contain information as all probabilities are ∼1/6. We can also see a resonant behavior in neuron 1, as there is a optimal level of noise ($∼10^{-5}$) for which the probabilities take most extreme values. This is consistent with the results presented in Ref. [12], where the spiking activity of a single FHN neuron (σ=0) was analyzed and it was shown that the patterns that are more or less expressed depend on the signal’s period. We also note that the noise level in neuron 2 does not have a significant impact in the encoding done by neuron 1: the signal is encoded (the probabilities computed from the spikes of neuron 1 take high or lower value) even when $D_{2}$ is large. This is due to the fact that the coupling strength is weak. The results for a higher coupling strength are displayed in Figure 7 (σ=0.01). We see the signal can still be encoded by neuron 1 when the level of noise is high, but it is transmitted to neuron 2 only when the level of noise in neuron 2 is not too large. For even higher coupling, in Figure 8 (ϵ=0.05) we see that the ordinal probabilities of both neurons are very similar, regardless of the noise level. This can be interpreted as due to the fact that the effect of the coupling is dominant with respect to the effect of noise. We point out however that, if the noise is not too strong ($D<10^{4}$) the over and less expressed patterns are due to the presence of the signal in neuron 1, because when $a_{0}=0$, as seen in Figure 9, all probabilities are ∼1/6. In contrast, if $D>10^{4}$ the combined effect of coupling and noise leads to the presence of over and less expressed patterns in the spike sequences. Therefore, this signal encoding mechanism is effective only if the noise and the coupling levels are weak enough, such that they do not generate, by themselves, over or less expressed patterns in the spike trains.

**Figure 10:**
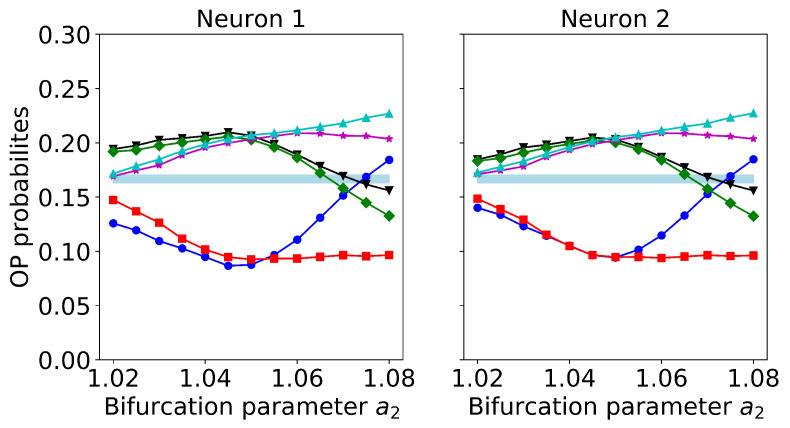
Effect of heterogeneous neurons’ parameters: we keep $a_{1}=1.05$ fixed and vary $a_{2}$. The left and right panels indicate the ordinal probabilities of neuron 1 and neuron 2, respectively. Other parameters are as in Figure 8.

Regarding differences in the neurons’s parameters, we found that they can either improve or degrade signal encoding and transmission. An example is presented in Figure 10, where we vary the parameter $a_{2}$, keeping $a_{1}=1.05$. We see that the encoding and transmission are degraded when $a_{2}<a_{1}$ because the ordinal probabilities approach the equi-probable region. A similar effect was found when changing the parameters $\epsilon_{1}$ and $\epsilon_{2}$ (not shown).

## 4. Discussion

We have used the FitzHugh–Nagumo model to simulate the activity of two coupled neurons when a weak periodic signal is applied to one of them. We have considered a signal that is below the spike-firing threshold; however, neural noise triggers spikes that encode and propagate the information of the weak signal. To investigate how the signal information can be encoded we have used symbolic ordinal analysis to uncover temporal structures in the timing of the spikes. We have found that if the noise level is not too strong, the spike trains of both neurons have over expressed and less expressed patterns, which depend on the features of the signal (the amplitude and the period).

The ordinal method used here has the main advantage that it uncovers patterns in sequences of spikes, but it has two main limitations: it does not consider amplitude information (i.e., the actual values of the intervals between spikes are disregarded) and suffers from finite size effects because long sequences of spikes are needed for a reliable estimation of the frequencies of occurrence of the ordinal patterns. Regarding the first limitation, ordinal analysis can be complemented with the analysis of the distribution of the inter-spike-intervals (ISIs) and the instantaneous spike rates of both neurons. We have done a preliminary study in this direction by analyzing how the mean ISI of the two neurons varies with the signal’s amplitude and period. We found a small variation of the mean ISI; however, pattern 210 was found to be over-expressed or under-expressed depending on the signal’s period.

Regarding the second limitation, if the frequencies of occurrence of the patterns are estimated from the spike trains of individual neurons, the data requirements render the symbolic encoding mechanism very slow (because long spike sequences are needed). However, if the encoding is performed by a large neuronal ensemble, and the patterns’ frequencies are estimated from the spike trains of many neurons, the encoding mechanism can be very fast because just a few spikes per neuron can be enough to determine the frequencies of the different patterns. Ongoing work is devoted to analyze this encoding mechanism in modular neuronal ensembles.

While we have shown that the ordinal probabilities depend on the amplitude and on the period of the signal, further analysis is needed in order to demonstrate that the amplitude or the period can be reliably recovered from the analysis of the ordinal probabilities. A relevant question is how much information about the signal can actually be recovered from the symbolic patterns. Additionally, further work is needed to address the impact of noise, in particular when the spiking activity is almost periodic and the inter-spike intervals take very similar values.

## Figures and Tables

**Table 1:** Ordinal patterns for L=3.

LABEL	SYMBOL	RELATION
1	012	$I_{3}>I_{2}>I_{1}$
2	021	$I_{2}>I_{3}>I_{1}$
3	102	$I_{3}>I_{1}>I_{2}$
4	120	$I_{2}>I_{1}>I_{3}$
5	201	$I_{1}>I_{3}>I_{2}$
6	210	$I_{1}>I_{2}>I_{3}$
